# Impact of the Social and Natural Environment on Preschool-Age Children Weight

**DOI:** 10.3390/ijerph15030449

**Published:** 2018-03-05

**Authors:** Inga Petraviciene, Regina Grazuleviciene, Sandra Andrusaityte, Audrius Dedele, Mark J. Nieuwenhuijsen

**Affiliations:** 1 Department of Environmental Sciences, Vytautas Magnus University, K. Donelaicio str. 58, 44248 Kaunas, Lithuania; regina.grazuleviciene@vdu.lt (R.G.); sandra.andrusaityte@vdu.lt (S.A.); audrius.dedele@vdu.lt (A.D.); 2 Centre for Research in Environmental Epidemiology (CREAL), Barcelona Biomedical Research Park, Dr. Aiguader, 88, 08003 Barcelona, Spain; mark.nieuwenhuijsen@isglobal.org

**Keywords:** green spaces, maternal education, psychosocial stress, childhood overweight/obesity

## Abstract

*Background:* The complex impact of environmental and social factors on preschool children being overweight/obese is unclear. We examined the associations between the levels of green space exposure and the risk of being overweight/obese for 4–6 year-old children and assessed the impact of maternal education on these associations. *Methods:* This cross-sectional study included 1489 mother-child pairs living in Kaunas, Lithuania, in 2012–2013. We assessed children overweight/obesity by standardized questionnaires using international body mass index cut-off points, and the level of greenness exposures by satellite-derived normalized difference vegetation index (NDVI) of each child’s home and by the distance to a nearest city park. The maternal education was used as the SES indicator. We used logistic regression models to investigate the strength of the associations. *Results:* Children from families with poorer maternal education, pathological mother-child relations and smoking mothers, and living in areas with less greenness exposure (NDVI-100 m), had significantly higher odds ratios of being overweight/obese. Lower maternal education and distance to a city park modified the effect of greenness cover level exposure on the risk of children being overweight/obese. *Conclusions:* Higher greenness exposure in the residential settings has beneficial effects on children’s physical development. The green spaces exposures for psychosocial stress management is recommended as a measure to prevent overweight/obesity among children.

## 1. Introduction

The increasing prevalence of being overweight/obese among children is a growing concern worldwide. In the European countries, the prevalence rates of overweight range from 8% to 30%, and obesity—from 1% to 13% among preschool-age children [1]. The prevalence rates of overweight/obesity were higher in Southern Italy (31.5%), Spain (29.4%) and Ireland (27.7%), while the lower rates were in Lithuania (9.6%) and Germany (11.9%). In turn, the prevalence remains very low in most developing countries, especially those in Asia and Africa (overweight < 5%, obesity < 2%), while in China and Brazil have seen a fast increase in the obesity problem in children population [2]. Childhood obesity has adverse social and health outcomes in children. Obese children are at a high risk of developing diabetes, cardiovascular disease, and the metabolic syndrome [3] as well as psychological problems in later life [4].

The underlying pathophysiology of childhood obesity is not fully clear, but there is some evidence suggesting that behavioral, genetic, and environmental factors play a role [5]. In the past decade, only few studies have examined associations between urban green spaces and obesity in preschool-age children, and findings were inconsistent [6,7,8,9]. These inconsistencies might reflect variation in health influences related to differences in the residential greenness exposure (e.g., parks proximity, residential surrounding greenness level) and variation in possibility to control for impact of possible confounding variables. The findings in New York City showed that physical activity and weight of urban preschool children from low-income families were associated with the environment near the home [10]. Limited research of young children suggests that living closer to city parks promotes physical activity, decreases sedentary behavior, improves motor coordination, decreases being overweight and improves children general health [11,12,13]. However, individual child and parents’ behavior and social environment may also have effect on the observed associations between green space exposures and being overweight in preschool aged children [14,15].

Recent studies have reported that chronic stress through health-related behavioral pathways in early childhood [16,17] and biological processes underlying predisposition to obesity [18] may have an impact on children being overweight. Some associations between parental education, health-related behaviors and children’s health have been reported [19,20]. However, very few studies have addressed the association between parent-child interaction and preschool-age child’s physical development [21,22,23]. One study did not find a difference in mother-child relationships between overweight and non-overweight children [24], while others have reported that dysfunctional parent-child interaction more often exists in families with obese children [25,26].

The available young children studies used different measures to assess environmental exposures and separately analysed the impact of the psychosocial environment and residential greenness levels and home proximity to city parks [6,9,21,22]. Meanwhile, these studies are very important because young children differ from adults in their physiological response, and if they do not get the environmental stimuli in early life stage to jumpstart their developmental potential then they may have the health problems later in life [27,28].

To our knowledge, the current study is one of the first to use a large sample of mother-child pairs to explore the influence of the joint effect of residence proximity to urban parks and greenness levels around the child’s home and SES on the risk of being overweight/obese in preschoolers by using objective greenness exposure levels measurements and controlling for possible confounding variables. The aim of this study was to examine whether the associations between quantified exposure to greenness level, defined as the vegetation index (NDVI) of the surroundings of a child’s residence, and a risk for being overweight/obese among 4−6 year-old children, are modified by the distance to a city park, and maternal education. Based on the previous studies [10,29], we hypothesized that children living in areas with less greenness exposure, and whose mothers with poorer education are at elevated risk of being overweight/obese. 

## 2. Materials and Methods

### 2.1. Study Population

This cross-sectional study is based on the population-based follow-up study of pregnant women who were recruited in 2007–2009 in the city of Kaunas, Lithuania. This study was carried out as part of the Positive Health Effects of the Natural Outdoor Environment in Typical Populations in Different Regions in Europe (PHENOTYPE) project funded by the European Commission Seventh Framework Program [30]. In 2012–2013, postal letters with an invitation to participate in the study, questionnaires, and informed consent forms were sent to 3292 mothers and their 4–6 year-old children using their home addresses known since pregnancy. Filled out postal questionnaires on health behavior, children’s health since birth, and other data were received from 1489 mothers followed-up for 4–6 years. A detailed description of the Kaunas cohort study and a flow chart of the inclusion criteria have been provided previously [31]. All 1489 mothers provided written informed consents, and the study protocol was approved by the Lithuanian Bioethics Committee, resolution No. 6B-12-147. The study complies with the Declaration of Helsinki. 

### 2.2. Green Space Exposure Assessment 

The level of the neighborhood greenness cover was estimated using the Normalized Difference Vegetation Index (NDVI) obtained from the satellite imagery as Landsat 7 Enhanced Thematic Mapper Plus data (resolution 30 m × 30 m). The NDVI is a quantified indicator of greenness based on land surface reflection of visible (red) and near-infrared parts of the spectrum. It ranges between −1 (usually water) and 1 (dense, healthy, green vegetation) [32]. Using the image resolution 30 m × 30 m measurements on 27 June 2013, we generated the NDVI Kaunas city map and estimated the mean NDVI values for each of 1489 participant homes in three circular buffers zones: 100 m, 300 m, and 500 m. These variables were geocoded using the participants’ addresses. In the statistical analyses, we used NDVI measurement cut-off point by median (>median and ≤median) and in every of three circular buffers zones we assessed greenness cover levels exposure effects on children overweight, totally analyzing 6 exposure groups. 

Using Urban Atlas data for Kaunas, we estimated home proximity to city parks as a straight-line distance to boundaries of a nearest city park. In the analyses of joint NDVI and distance to a park effects on children overweight, we dichotomized the distance of the residence to a nearest city park into two greenness exposure categories: higher greenness exposure—distance below or equal to 300 m, and lower greenness exposure—more than 300 m distance to a park. The distance of 300 m radius was selected according to the recommendation of the European Commission for access to green spaces [33]. All Kaunas city parks (regarded >1 ha) are free accessible, without fence, open to the public, and have a similar infrastructure. Individual modelled exposure to ambient NO_2_ was assigned using the Land-use regression (LUR) model [34].

### 2.3. Measures of Overweight/Obesity

Individual data on socio-demographic characteristics, living environment factors, health behavior, nutrition, and other covariates were obtained using standardized questionnaires. The responses of the mother-child questionnaire were utilized to identify children who were overweight/obese, other physical health conditions, physical activity, and exposures. Information on height (in cm) and weight (in kg) was used to estimate children body mass index. The body mass index was calculated as the child’s weight (in kg) divided by height (in m^2^). We used the age group- and sex-fixed BMI cut-off points for the estimation of children’s overweight and obesity according to the Childhood Obesity Working Group of the International Obesity Taskforce (IOTF) guidelines [35,36]. Because of the small number of obese children (*N* = 36), overweight and obese groups were combined in the analysis, and referred to as being overweight/obese throughout the manuscript. We analyzed two BMI groups—the overweight/obesity group (defined as BMI ≥ 18 kg/m^2^) and the reference group (defined as BMI < 18 kg/m^2^).

### 2.4. Risk Factors for Overweight/Obesity 

Basing on questionnaire information, we selected potential predictors for being overweight/obese for estimating their impact on the studied associations. 

In the statistical analyses, a singleton infant’s birth weight in grams was dichotomized according to the mean of the cohort birth weight (≥3450 g and <3450 g). The evaluation of child physical inactivity (sedentary behavior) was based on answers to questions on the duration of television watching and computer use. The following questions were asked “How many hours per day does your child watch television on weekdays?” and “How many hours per day does your child spend at the computer on weekdays?” The responses were recorded as the mean number of hours of sedentary behavior per day. According to the recommendation of the American Academy of Pediatrics, the screen time should be limited to 1–2 h/day depending on the child’s age [37]. We calculated the distribution of study children by total sedentary behavior hours per day and estimated median hours. In our sample, the total median hours spend on the computer and TW was 3.0. Therefore, in the statistical analyses we used a binary variable indicating whether the child spent less or more than 3 h watching television or using a computer during each working day.

In this study, maternal education level was used as the SES indicator in the analysis [38]. Mothers reported their educational achievement on inclusion into the study: primary education and 10 or fewer schooling years were treated as a lower level of education, and more than 10 schooling years—as a higher level of education.

Questionnaire responses by parents or guardians were used to categorize information on tobacco smoke and other exposures. Maternal smoking during pregnancy and secondhand tobacco smoking were both dichotomized. 

To evaluate maternal self-reported mother-child relationship, we used the Parent-Child Dysfunctional Interaction subscale (PCDI) of the Parenting Stress Index Lithuanian short version form (S-PSI/SF). In this study, the internal consistency of the PCDI subscale, as measured by Cronbach‘s alpha, was 0.85 [39,40]. Three categorical variables were created by using percentiles as cut-off points: normal—below the 85th percentile, borderline—the 85th to the 90th percentile, and pathological mother-child relations—above the 90th percentile. The 85th percentile as a cut-off-point was sufficient for identifying exposure to maternal stress [41].

### 2.5. Statistical Analysis

The study covariates such as mother-child individual characteristics and exposures were summarized with descriptive statistics. We identified the risk factors for being overweight/obese using chi-squared and unadjusted logistic regression analysis and compared the distribution of the variables between children being overweight/obese (case group) and the referent group (children who are not overweight/obese). We used standard frequency tables for the estimation of the risk factors for children’s weight status by calculating odds ratios (OR) with their 95% confidence intervals (CI). Predictor variables whose univariate test showed a statistically significant association (*p* < 0.05) with the outcome or those that changed the adjusted odds ratios (aOR) by 10% or more were retained for inclusion in multivariable logistic regression analyses.

Logistic regression analyses were used to assess the effects of greenness exposures on the risk of being overweight/obese using both categorical (by median) and continuous (IQR, per 100 m increase) measures. We examined the unadjusted and adjusted relationships between greenness levels (NDVI) in circular 100 m, 300 m, and 500 m around the home and distance to a city park of a child’s home and children’s overweight/obesity status. A multivariate logistic regression was used to investigate the effects of greenness exposure (as adjusted OR and 95% CI) on being overweight/obese controlling for potential confounders. In a stratified analysis by greenness exposure, we also assessed the joint effect of living farther away from a nearest park (>300 m) and maternal education level on the risk of being overweight/obese among children. Odds ratios were adjusted for several known covariates that could affect the strength of the association between greenness exposures and children being overweight/obese: family status, maternal age, education, employment status, smoking during pregnancy, secondhand smoking, mother-child relations, NO_2_, the child’s sex, birth weight, and sedentary behavior. All statistical analyses were conducted with SPSS version 20.0 software (IBM Corporation, New York, NY, USA).

## 3. Results

Of the 3292 mother-child pairs who were invited to participate in this study, 1489 mothers (45.0%) provided a written informed consent and agreed to participate. The analyses of non-participants on the basis of the data available from at-birth questionnaires showed that the sociodemographic variables were similar and not statistically significantly different from those of the participants with regard to the family status, education level, other characteristics, smoking during pregnancy, or birth outcomes (data not shown). 

The descriptive statistics of all of the participants’ characteristics that were used in the analyses are presented in Table 1. The group of better-educated mothers was statistically significantly larger than the group of less educated mothers. Smoking during pregnancy was reported by 7.6% of mothers, and 35.8% were exposed to secondhand tobacco smoke. In about 51.9% of the mothers, the birth weight of their newborn was ≥3450 g, and 34.1% of mothers had borderline or pathological relations with their child. 

The mean age of the children at the time of the investigation was 4.7 years (SD = 0.8). The BMI cut-off points based on the International Obesity Taskforce classifications indicated that 7.5% of the children were overweight/obese (BMI ≥ 18 kg/m^2^).

The averages (minimum and maximum) for the quantified indicator of greenness levels (NDVI) within 100 m, 300 m, and 500 m buffers of the residence were 0.544 (0.189–0.821), 0.548 (0.290–0.830), and 0.553 (0.335–0.805), respectively. About 25.7% of the participants’ homes were within the distance of 300 m from the nearest city park. 

Table 2 presents unadjusted and adjusted associations between the participants’ characteristics and children’s overweight/obesity status. In an unadjusted analysis, statistically significant differences in prevalence of some characteristics and higher odds ratios were found for the overweight/obese children compared to the referent group: maternal lower education, smoking during pregnancy, pathological mother-child relations, the child’s male sex, higher than mean birth weight (≥3450 g), and sedentary behavior more than 3 h per day. We treated these covariates as possible determinants of children being overweight/obese. After adjustment, the odds ratios for being overweight/obese remained statistically significant higher for children of lower educated mothers, exposed to tobacco smoke, pathological mother-child relations, higher birth weight, and sedentary behavior. 

The prevalence of overweight/obesity among children residing in areas with higher greenness (NDVI-100 m > median) was 5.9%, while in the areas with lower greenness (<median), the prevalence was 9.0%. However, a statistically significant association between greenness exposure and children being overweight/obese was only observed for the 100-m buffer size with adjusted OR of 1.72, 95% CI 1.15–2.60 (the median or below vs. above the median); also an increase in the IQR of green space was associated with a 43% increase in the risk of being overweight/obese, after adjustment for covariates. An increase in the distance to a city park beyond 300 m from the child’s home had a tendency to increase for the risk of children being overweight/obese, but the result was not statistically significant.

Table 3 provides unadjusted and adjusted associations between the greenness exposure and children being overweight/obese. With reference to the group of high greenness exposure (NDVI-100 m > median and the distance to a city park ≤300 m), a greater distance to a park tended to increase the risk of overweight/obesity among children in unadjusted (by 26%) and multivariate-adjusted (by 28%) models. The prevalence of being overweight/obese was higher among children residing in areas with low greenness exposure (NDVI-100 m ≤ median and the distance to a city park >300 m), and was associated with a statistically significantly increased risk of being overweight/obese in 4–6 year-old children. The OR was 2.27 (95% CI 1.12–4.62) after adjustment for family status, maternal age, education, smoking during pregnancy, secondhand smoking, employment status, mother-child relations, NO_2_, the child’s sex, birth weight, and sedentary behavior. The results showed that further distance to a city park strengthened the lower greenness level exposure effect on the increased risk of young children being overweight/obese.

To study the combined effects of greenness exposure and maternal education level, we simultaneously evaluated the risks associated with lower education, NDVI-100 m and overweight/obesity among 4–6 year-old children. Table 4 provides the results of univariate and multivariable models stratified by greenness level (NDVI index) and maternal education level. With reference to the group of NDVI-100 m > median and higher maternal education, a lower exposure to greenness statistically significantly increased the risk of being overweight/obese in 4–6 year-old children in both unadjusted and adjusted models. Overweight/obesity rates were also higher for children with lower maternal education. However, for children residing in areas with low exposure to greenness, and lower educated mothers the odds ratios were much higher. The OR was 3.18, 95% CI 1.65–6.13, after adjustment for family status, maternal age, smoking during pregnancy, secondhand smoking, employment status, mother-child relations, the child’s sex, birth order, birth weight, and sedentary behavior. The results showed that lower maternal education strengthened the effect of low exposure to greenness on the increased risk of young children being overweight/obese.

## 4. Discussion

The results of this epidemiological study strengthen the evidence of the impact of residential quantified green space exposure and social behavioral factors influencing of the risk of being overweight/obese in 4–6 year-old children. In addition, our findings revealed that further distance to a city park and lower maternal education strengthened the effect of low exposure to greenness on the increased risk of young children being overweight/obese. This study found evidence that higher NDVI level exposure is associated with reduced the risk of being overweight/obese and that SES and home distance to parks modify this relationship.

The unfavorable social behavioral and low residential greenness that increased the risk of being overweight/obese in 4–6 year-old children were the following: lower maternal education, pathological mother-child relations, maternal smoking during pregnancy, the child’s sedentary behavior, and lower residential greenness exposure (NDVI-100 ≤ median). These variables have statistically significant impact on the prevalence of overweight/obesity among young children. Our study results supported the hypothesis that children living in lower greenness exposure areas or whose mothers with poorer education are at elevated risk of being overweight/obese. 

The results of our study are in accordance with those of studies performed in other countries that found associations between unfavorable early-life factors, such as poorer maternal education or tobacco smoking and childhood overweight/obesity in later life [42,43,44]. There is speculation that less years of schooling for the mothers result in lower income and decrease the capacity to incorporate health recommendations [45], meanwhile, some studies did not find social differences in the risk of being overweight/obese [46]. The impact of intrauterine tobacco exposure might be due to mediating effect by socioeconomic status. The tobacco smoking is associated with the toxicity nicotine and carbon monoxide whose depress the development of the immune system in fetus and disturb adipocytes tissue hormone leptin activity which can result in metabolic disorders and obesity in later life [47,48]. The mechanism whereby in prenatal smoking can produce heritable changes in obesity has been suggested to be due to the DNA methylation [49].

This study results are in accordance with the results from the other studies suggesting the associations between maternal stress or low-SES and preschool children being overweight [50,51,52]. Previous studies included different psychometric characteristics such as family functioning, parenting style, stressful life events, distress and none of them took into measures parent-child interactions. Thus, our study expands previous knowledges about parent-child relations and overweight/obesity in preschoolers [50,53]. However, Brødsgaard et al. found a non-statistically significantly impact of dysfunctional mother-child relations (by the prototypes) on young child BMI due to a small number of subjects or culture aspects [24]. 

The poor quality of parent-child interactions may influence the risk for children being overweight/obese through biological and behavioral pathways. The family-level stressors (e.g., family divorce, parent-child conflict, poor quality relations) are related to increased secretion of the stress hormones (mainly cortisol and catecholamine) in the child‘s body, which can result in visceral obesity [26]. From a behavioral point of view, parenting psychosocial stress may influence the development of childhood obesity due to the child‘s emotional eating, poor-sleep quality, and family role in promoting sedentary lifestyles [54].

Our results showed that sedentary behavior is one of the independent risk factors for being overweight/obese in children. These data are in line with previous research, showing that being overweight is associated with lifestyle factors, including sedentary behavior [55]. Additionally, the cohort study carried out in New Zealand concluded that low levels of physical activity (sedentary behavior) measured by accelerometers increased the likelihood of the child or teen being overweight and obese [56].

Our findings on the beneficial effect of greenness on preschool-age children risk of being overweight/obese are consistent with those previous studies [7,9]. Based on the findings of cohort studies, the prevalence of overweight/obesity was less likely to be observed in children residing in areas with higher neighborhood greenness exposure [7] and near a city park [57]. The findings of a study conducted in Indiana showed that greater amounts of vegetation around preschool-age children’s residences were associated with a reduced body weight, but only among those in areas with greater population density [9]. However, in Turkey green space was not associated with young children’s overweight. It is possibly due to neighborhood greenness insufficient design, lack of certain park facilities and parental concerns about safety [6]. In this study, the effects of green spaces on preschool-age children’s risk of overweight/obesity depended on both the surrounding greenness level in the residential areas and the distance of the residences to city parks. Our study findings showed that lower residential surrounding greenness level increase the risk of being overweight/obese in 4–6 year-old children and residence distance to a city park might modify this effect.

In this study we were also able to show that maternal education mediated the relationship between level of greenness and childhood risk of being overweight/obese (aOR 3.18; 95% CI 1.65–6.13). These data are in line with the findings of Schalkwijk et al. who found that less greenness exposure of children of low SES families is associated with increased risk of being overweight in preschool children [29]. A study conducted in New York City reported that a higher density of street trees rather than the park area was associated with a lower prevalence (by 12%) of obesity in low-income families [10]. However, in a previous study of urban low-income preschoolers aged 3–5 years, being overweight in childhood was not associated with proximity to park playgrounds [8]. 

To our knowledge, this study was one of the first to demonstrate an interaction between quantified neighborhood greenness levels as measured by the vegetation index (NDVI), residential distance to a city park, SES and the risk of being overweight/obese in 4–6 year-old children. The observed effect modification showed that the risk of being overweight/obese in children are higher for children residing in areas with low greenness exposure and mothers of lower SES. 

The underlying mechanisms for the positive impact of the surrounding greenness on children’s health may be partially explained by the findings of a physiological studies showing that contact with nature has the potential to improve oxygenation and metabolism by reducing psychophysiological stress and decreasing the excretion of catecholamine [58]. In addition, green environment can facilitate homeostasis through its positive effects on the central and autonomic nervous system, the endocrine system, and cardiovascular system [59].

Some studies reported that the neighborhoods that have more green spaces are associated with such parents and their children behavior as outdoor play or physical activity [60] and physical activity is positively associated with green space quality and access to parks [12]. In turn, it needs less for parents to transportation their children to other physical activity facilities [60]. The studies that used GPS units and accelerometers in children, found that about half of weekend moderate-vigorous physical activity took place in green space [61], and that moderate physical activity were significantly more likely intense in green space for boys, but not for girls [62]. Moreover, green space may promote mental health by encouraging physical activity, providing a direct psychological benefit or social cohesion [63]. Sugiyama et al. noted that the neighborhood greenness was associated with better mental health [64]. However, in a Lithuanian study, Balseviciene et al. found that living closer to city parks was associated with improved mental health in children whose mothers had a lower education level [65].

The main strengths of this study include the big sample size, the quantified estimations of greenness exposures on the individual level, use of psychologically valid instruments to measure dysfunctional mother-child relations and the ability to control for a large range of potential overweight/obesity confounders. Additional strengths include the possibility of avoiding exposure misclassification associated with the participants’ mobility and the ability to assess the impact of sedentary behavior on the association between greenness exposure and risk of being overweight/obesity in children’s. However, this study also has some limitations. Data on the children’s height and weight were obtained from parental self-report through a questionnaire, which might have resulted in misclassification of health outcomes and might have attenuated the strengths of the observed associations. In this study, the proximity to city parks was assessed as a straight line from the child’s residential address to a city park without conducting the analysis of direct accessibility, and the measure of the NDVI only quantified the amount of green space in a residential area and could not account for the type or quality of the green space. Nevertheless, both measures are objective; NDVI presents the level of surrounding greenness but does not distinguish between grass field and trees. We have no data for usage of green spaces and time spent in the park or in green places near the home or farther away from the home. In addition, this study did not include measures linked to family style and functioning, stressful life events, family-level stressors, nutrition, weight gain during pregnancy, and genetic factors, that might have affected the obtained results. However, these limitations are assumed random.

## 5. Conclusions

The findings of the present study complement the knowledge base with the impact of residential quantified green space exposure and social behavioral factors on overweight/obesity in 4–6 year-old children, controlling for important covariates. This study found evidence that higher NDVI level exposure is associated with reduced the risk of being overweight/obese and that maternal education and home distance to parks modify this relationship. There is need for preventive health program that encourage using the green spaces, focusing particularly on lower educated mothers of children. Therefore, the green spaces exposures for psychosocial stress reduction has been recommended as a measure to prevent overweight/obesity among children.

Future studies should continue to investigate the impact of environmental, modifiable lifestyle factors, family stressors, and genetic factors leading to susceptibility to obesity. Additionally, further studies are needed to estimate the impact of the quality of greenery on park usage, the time spent in the natural environmental, and children’s physical health. 

## Figures and Tables

**Table 1:** Characteristics of study participants.

Characteristics	Mean (SD) or *N* (%)
Child’s age (years)	4.7 (0.8)
Height (cm)	110.6 (7.8)
Weight (kg)	18.8 (3.7)
BMI (kg/m^2^)	15.3 (1.9)
Overweight/obesity (kg/m^2^)	111 (7.5)
Child’s sex	
Female	751 (50.4)
Male	738 (49.6)
Birth order	
First child	822 (55.2)
2nd and later	667 (44.8)
Birth weight	
<3450 g	716 (48.1)
≥3450 g	773 (51.9)
Sedentary behavior	
≤3 h per day	1174 (78.8)
>3 h per day	315 (21.2)
Maternal age at childbirth	
≤30 years	995 (66.8)
>30 years	494 (33.2)
Maternal education	
Lower (10 or less years)	1182 (79.4)
Higher (>10 years)	307 (20.6)
Family status	
Both parents	1293 (86.8)
Single mother	196 (13.2)
Employment status	
Unemployed	396 (26.7)
Employed	1091 (73.3)
Breastfeeding	
No	99 (6.6)
Yes	1390 (93.4)
Smoking during pregnancy	
No	1376 (92.4)
Yes	113 (7.6)
Secondhand smoking	
No	956 (64.2)
Yes	533 (35.8)
Mother-child relations	
Normal	980 (65.9)
Borderline	419 (28.1)
Pathological (abnormal)	90 (6.0)
Residential annual greenness level (NDVI)	
NDVI-100 m buffer	0.544 (SD: 0.09; range: 0.189–0.821)
NDVI-300 m buffer	0.548 (SD: 0.08; range: 0.290–0.830)
NDVI-500 m buffer	0.553 (SD: 0.08; range: 0.335–0.805)
Ambient NO_2_ (µg/m^3^)	15.99 (SD: 2.62; range: 8.85–31.05)
Distance to a city park	
≤300 m	382 (25.7)
>300 m	1107 (74.3)

**Table 2:** Unadjusted and adjusted associations between the participant’s characteristics and children’s overweight/obesity.

Characteristics	Referent *N* (%)	Overweight *N* (%)	Unadjusted	Adjusted ^†^
			OR 95% CI	
Maternal education				
Higher (>10 years)	1107 (93.7)	75 (6.3)	1	1
Lower (10 or less years)	271 (88.3)	36 (11.7)	1.96 * (1.29–2.98)	1.80 * (1.12–2.90)
Family status				
Both parents	1197 (92.6)	29 (7.3)	1	1
Single mother	182 (92.0)	82 (7.5)	1.09 (0.61–1.96)	0.85 (0.46–1.58)
Maternal age at childbirth				
≤30 years	462 (93.5)	32 (6.5)	1	1
>30 years	916 (92.10)	79 (7.9)	1.25 (0.81–1.91)	1.21 (0.75–1.94)
Employment status				
Unemployed	369 (92.7)	29 (7.3)	1	1
Employed	1009 (92.5)	82 (7.5)	1.03 (0.67–1.61)	0.77 (0.49–1.23)
Smoking during pregnancy				
No	1282 (93.2)	94 (6.8)	1	1
Yes	96 (85.0)	17 (15.0)	2.42 * (1.38–4.21)	2.05 * (1.13–3.72)
Secondhand smoking				
No	888 (92.9)	68 (7.1)	1	1
Yes	490 (91.9)	43 (8.1)	1.15 (0.77–1.71)	0.86 (0.56–1.32)
Mother-child relations				
Normal	908 (93.3)	65 (6.7)	1	1
Borderline	390 (91.9)	34 (8.1)	1.22 (0.79–1.88)	1.09 (0.70–1.71)
Pathological (abnormal)	80 (86.7)	12 (13.3)	2.13 * (1.10–4.11)	1.93 * (1.00–3.80)
Breastfeeding				
Yes	1289 (92.7)	101 (7.3)	1	1
No	89 (89.9)	10 (10.1)	1.43 (0.72–2.84)	0.84 (0.42–1.72)
Child’s sex				
Male	705 (93.9)	46 (6.1)	1	1
Female	673 (91.2)	65 (8.8)	1.48 (1.00–2.19)	1.46 (0.98–2.18)
Birth order				
First child	761 (92.6)	61 (7.4)	1	1
2nd and later	617 (92.5)	50 (7.5)	1.01 (0.69–1.49)	0.98 (0.63–1.52)
Child’s birth weight				
<3450 g	674 (94.1)	42 (5.9)	1	1
≥3450 g	704 (91.1)	69 (8.9)	1.57 * (1.06–2.34)	1.57 * (1.05–2.36)
Sedentary behavior				
≤3 h per day	1111 (93.7)	75 (6.3)	1	1
>3 h per day	267 (88.2)	36 (11.8)	1.98 * (1.30–3.03)	1.77 * (1.15–2.71)
Natural environment ^††^				
NDVI-100 m				
>median	700 (94.1)	44 (5.9)	1	1
≤median	678 (91.0)	67 (9.0)	1.57 * (1.16–2.33)	1.72 * (1.15–2.60)
IQR ^‡^			1.45 * (1.10–1.85)	1.43 * (1.10–1.91)
NDVI-300 m				
>median	692 (92.9)	53 (7.1)	1	1
≤median	686 (92.2)	58 (7.8)	1.10 (0.75–1.63)	1.13 (0.76–1.67)
IQR ^‡^			1.17 (0.89–1.85)	1.15 (0.91–1.96)
NDVI-500 m				
>median	693 (93.4)	49 (6.6)	1	1
≤median	685 (91.7)	62 (8.3)	1.28 (0.93–2.48)	1.32 (0.89–1.97)
IQR ^‡^			1.22 (0.84–1.88)	1.19 (0.82–1.91)
Distance to a city park				
≤300 m	361 (94.5)	21 (5.5)	1	1
>300 m	1017 (91.9)	90 (8.1)	1.52 (0.93–2.48)	1.51 (0.92–2.49)
Continuous variable (per 100 m increase)			1.01 (0.97–1.04)	1.01 (0.98–1.05)

* *p*-value < 0.05. ^‡^ IQR increase. ^†^ Adjusted for: family status, maternal age at childbirth, education, smoking during pregnancy, secondhand smoking, employment status, mother-child relations, breastfeeding, the child’s sex, birth order, birth weight, and sedentary behavior. ^††^ Adjusted for: family status, maternal age at childbirth, education, smoking during pregnancy, secondhand smoking, employment status, mother-child relations, NO_2_, the child’s sex, birth weight, and sedentary behavior.

**Table 3:** Joint effect of greenness cover level and home distance to the nearest city park on the risk of children’s overweight/obesity (stratified analysis).

Greenness Exposure Level	Referent *N* (%)	Cases *N* (%)	Unadjusted OR 95% CI	Adjusted ^†^ OR 95% CI
NDVI-100 > median & distance to a city park ≤300 m	189 (95.0)	10 (5.0)	Referent 1	Referent 1
NDVI-100 > median & distance to a city park >300 m	511 (93.8)	34 (6.2)	1.26 (0.61–2.60)	1.28 (0.61–2.67)
NDVI-100 ≤ median & distance to a city park ≤300 m	172 (94.0)	11 (6.0)	1.21 (0.50–2.92)	1.36 (0.56–3.34)
NDVI-100 ≤ median & distance to a city park >300 m	506 (90.0)	56 (10.0)	2.09 * (1.05–4.18)	2.27 * (1.12–4.62)

* *p*-value < 0.05. ^†^ Adjusted for: family status, maternal age at childbirth, education, smoking during pregnancy, secondhand smoking, employment status, mother-child relations, NO_2_, the child’s sex, birth weight, and sedentary behavior.

**Table 4:** Association between residential greenness level (NDVI-100 m radius), maternal education, and children’s overweight/obesity (stratified analysis).

Greenness Exposure & Maternal Education	Referent *N* (%)	Cases *N* (%)	Unadjusted OR 95% CI	Adjusted ^†^ OR 95% CI
NDVI-100 > median & higher education	546 (95.3)	27 (4.7)	1	1
NDVI-100 ≤ median & higher education	561 (92.1)	48 (7.9)	1.73 * (1.06–2.81)	1.80 * (1.10–2.95)
NDVI-100 > median & lower education	154 (90.1)	17 (9.9)	2.23 * (1.19–4.20)	2.02 * (1.03–4.00)
NDVI-100 ≤ median & lower education	117 (86.0)	19 (14.0)	3.28 * (1.77–6.10)	3.18 * (1.65–6.13)

* *p*-value < 0.05. ^†^ Adjusted for: family status, maternal age at childbirth, maternal smoking during pregnancy, secondhand smoking, employment status, mother-child relations, the child’s sex, birth order, birth weight, and sedentary behavior.
